# Divinylpyrimidine reagents generate antibody–drug conjugates with excellent *in vivo* efficacy and tolerability†

**DOI:** 10.1039/d1cc06766d

**Published:** 2022-01-13

**Authors:** Stephen J. Walsh, Soleilmane Omarjee, Friederike M. Dannheim, Dominique-Laurent Couturier, Dorentina Bexheti, Lee Mendil, Gemma Cronshaw, Toby Fewster, Charlotte Gregg, Cara Brodie, Jodi L. Miller, Richard Houghton, Jason S. Carroll, David R. Spring

**Affiliations:** Yusuf Hamied Department of Chemistry, University of Cambridge, Lensfield Road Cambridge CB2 1EW UK spring@ch.cam.ac.uk; Cancer Research UK Cambridge Institute, University of Cambridge, Robinson Way Cambridge CB2 0RE UK Jason.carroll@cruk.cam.ac.uk; MRC Biostatistics Unit, University of Cambridge UK

## Abstract

The development of divinylpyrimidine (DVP) reagents for the synthesis of antibody–drug conjugates (ADCs) with *in vivo* efficacy and tolerability is reported. Detailed structural characterisation of the synthesised ADCs was first conducted followed by *in vitro* and *in vivo* evaluation of the ADCs’ ability to safely and selectively eradicate target-positive tumours.

## Abstract 2

The development of divinylpyrimidine (DVP) reagents for the synthesis of antibody–drug conjugates (ADCs) with *in vivo* efficacy and tolerability is reported.

## Graphical abstract

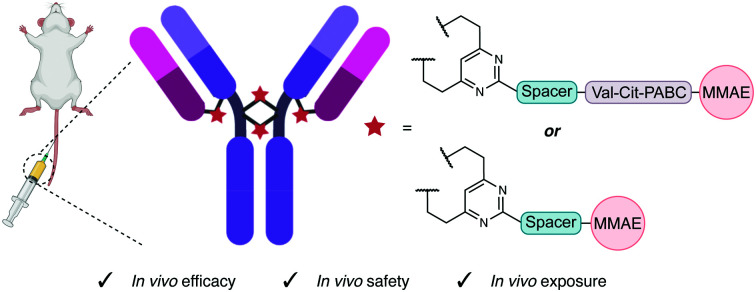

Recent years have witnessed a resurgence in the clinical success of ADCs. Eleven ADCs have now obtained regulatory approval from the US Food and Drug Administration, with >100 others currently undergoing clinical investigation.^1^ These unique modalities have long promised the potential of precise anti-cancer therapy due to their unique ability to selectively deliver highly potent cytotoxins to cancer cells. While the antibody and payload are of critical importance to the pharmacology, the bioconjugation linker is also crucial to ensure clinical success.^2^ Early generation ADCs focused on stochastic modification of lysine or interchain cysteine residues, generating a highly heterogeneous mixture of species in each synthetic batch of ADC. The focus has now shifted toward site-selective modification strategies that enable precise installation of a desired number of payloads at specific, predictable sites.^3,4^ Obtaining perfectly homogeneous ADC products with these technologies remains challenging but they undoubtedly generate a less heterogeneous mixture than early generation bioconjugation approaches. Many strategies have been successfully employed in this endeavour, including the genetic introduction of additional cysteine residues,^5^ reactive recognition tags^6^ or unnatural amino acids,^7,8^ remodelling of the glycan chains^9^ or enzymatic modification of conserved amino acid sequences.^10^ Another strategy that has shown early stage promise is disulfide rebridging.^11^ Reduction of the 4 interchain disulfides in a human(ised) IgG1 is followed by treatment with a thiol-selective bis-reactive linker that can covalently cross-link the reduced cysteines. Modification in this way controls the site of modification, the number of drug molecules attached (typically 4) and can increase the stability of the bioconjugate by reforming covalent bonds between the light and heavy polypeptide chains. Several disulfide rebridging reagents have been reported for the synthesis of ADCs including pyridazinediones,^12^ next-generation maleimides,^13^ bissulfones,^14^ arylene dipropiolonitriles,^15^ DiPODS,^16^ diethynylphosphinates^17^ and chloroacrylates,^18^ amongst others. We have previously reported the use of DVP^19,20^ and divinyltriazine (DVT)^21^ reagents, which have been shown to generate exceptionally stable ADCs, and which are compatible with a wide variety of payloads.^22,23^ Herein, we conduct further structural characterisation of both cleavable and non-cleavable ADCs synthesised *via* DVP bridging. *In vitro* and subsequent *in vivo* investigation of these ADCs demonstrate their ability to be utilised in either construct to generate safe and efficacious ADCs.

To commence investigations, non-cleavable DVP-PEG_4_-MMAE 1 and cleavable DVP-PEG_4_-Val-Ala-PABC-MMAE 2 were synthesised (see ESI† for synthetic details). Next, synthesis of the desired ADCs was undertaken. For this proof-of-concept study, the anti-HER2 antibody trastuzumab was chosen as a model antibody due to its widespread use in clinical and marketed ADCs. The interchain disulfides in trastuzumab were first reduced by treatment with tris(2-carboxyethyl)phosphine hydrochloride (TCEP), followed by treatment with either DVP 1 or 2 (Fig. 1). LCMS and SDS-PAGE analysis revealed conversion to the desired cleavable (C-ADC) and non-cleavable (NC-ADC) ADCs (ESI,† Fig. S1–S3). While SDS-PAGE and LCMS analysis suggested good bridging efficiency, a quantitative determination of the precise drug–antibody ratio (DAR), and the variability therein, was desired. Hydrophobic interaction chromatography (HIC) analysis of both ADCs revealed that the major species in both ADCs was the desired DAR 4 construct, with minor amounts of DAR 3 and DAR 5 species observed in both ADCs (Fig. 2a and b). The average DAR values were measured at 3.91 and 3.89 for the cleavable and non-cleavable ADCs, respectively. The introduction of hydrophobic payloads, such as MMAE, can increase the aggregation propensity of an antibody. It is preferable that any modification of an antibody does not cause significant protein aggregation as this can detrimentally affect the pharmacokinetic profile of the administered ADC, and thus its overall pharmacology. To investigate if DVP-mediated installation of *ca.* 4 cleavable or non-cleavable MMAE moieties onto trastuzumab significantly affects the antibody's monomer content, the ADCs were analysed by size-exclusion chromatography (SEC). Both ADCs had highly similar SEC traces compared to unmodified trastuzumab with >97% monomer observed in each case, indicating that our DVP-based ADCs were not prone to excessive aggregation (Fig. 2c). Next, to evaluate the effect of modification on the antibody's affinity for HER2, an enzyme-linked immunosorbent assay (ELISA) was performed. Both ADCs displayed similar binding affinity for HER2 compared to unmodified trastuzumab (Fig. 2d), indicating that modification with the bulky linker-payloads did not negatively affect receptor binding (in this *in vitro* assay). Due to the different active metabolite released from the cleavable and non-cleavable ADCs, it was hypothesised that they would differ in their ability to kill their target cells. As such, a direct comparison of the ADCs by treatment of two HER2-positive (SKBR3 and BT474) and two HER2-negative (MCF7 and MDA-MB-468) breast cancer cell lines was conducted (Fig. 2e and f). Indeed, both ADCs displayed dose-dependent cytotoxicity against both HER2 cell lines with the cleavable ADC being slightly more potent. Both ADCs showed excellent selectivity over HER2-negative cells with cytotoxicity only observed above 100 nM in all cases. The similarity between the cleavable and non-cleavable MMAE ADCs in HER2-positive cancer cell lines has been previously reported.^24^

**Fig. 1:**
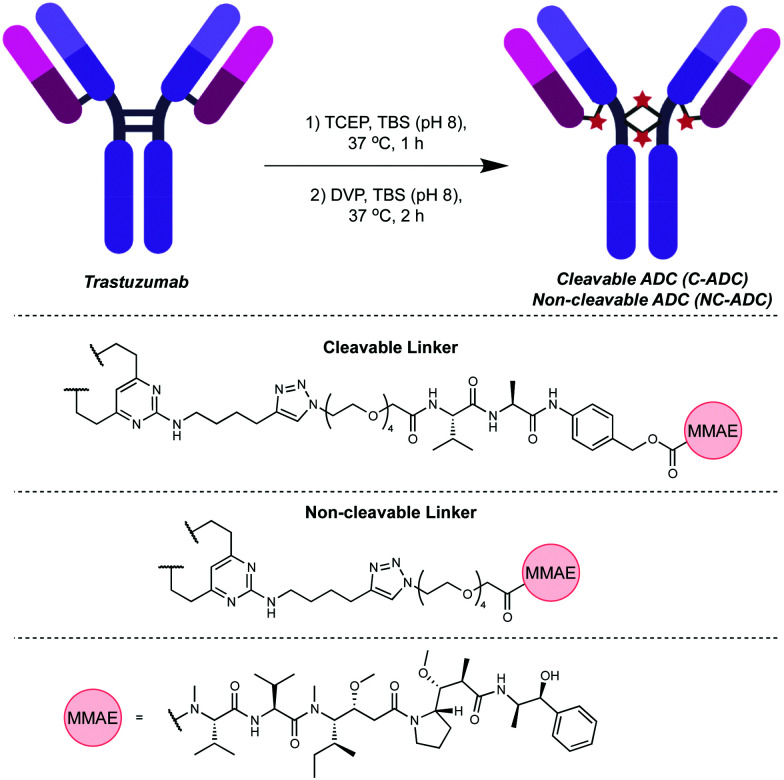
Modification of trastuzumab with DVP reagents generates either a non-cleavable or cleavable ADC. TBS = Tris-buffered saline (25 mM TrisHCl, 25 mM NaCl, 0.5 mM EDTA (pH 8)).

**Fig. 2:**
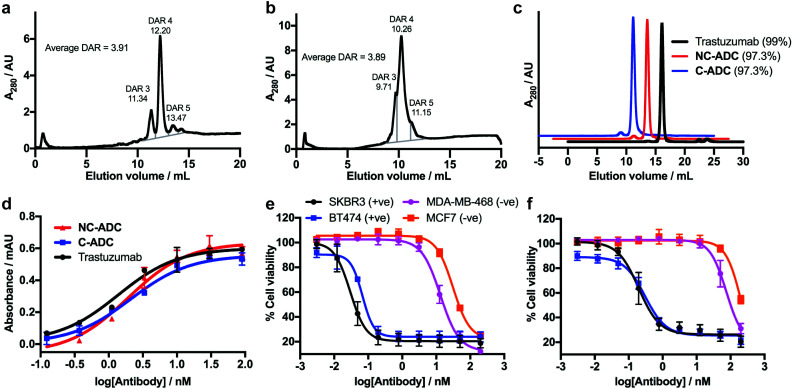
*In vitro* characterisation of DVP ADCs. (a) HIC trace of C-ADC, (b) HIC trace of NC-ADC, (c) SEC traces of trastuzumab, NC-ADC and C-ADC, (d) HER2 ELISA of trastuzumab, NC-ADC and C-ADC. Error bars represent the standard deviation of biological quadruplicates, (e) cell viability of C-ADC against HER2-positive (+ve) and HER2-negative (−ve) cell lines, and (f) cell viability of NC-ADC against the same cell lines. Viability data shows the mean of three independent replicates and error bars represent S.E.M.

The ability of the ADCs to inhibit tumour growth *in vivo* was then investigated in a BT474 breast cancer xenograft model.^25,26^ The ADCs were administered *via* tail vein intravenous (IV) injection into NSG mice bearing subcutaneous tumour xenografts on their right flank. Mice were enrolled into treatment arms once tumour volume reached 200 mm^3^, with each mouse receiving two, weekly doses of the C-ADC, NC-ADC, trastuzumab or vehicle. To evaluate the efficacy and tolerability of the ADCs, each ADC was split into three treatment arms, with mice in each cohort receiving 1, 10 or 20 mg kg^−1^ ADC based on total antibody content and their bodyweight the day prior to administration. At 1 mg kg^−1^, there was no statistically significant tumour growth inhibition compared to treatment with vehicle (Fig. 3a, b and ESI,† Tables S1, S2). In contrast, both ADCs showed complete tumour regression at either 10 or 20 mg kg^−1^ for up to 60 days after first administration. While trastuzumab (10 mg kg^−1^) showed partial tumour regression immediately after treatment, upon cessation of treatment, tumour growth rate was not significantly different to vehicle (ESI,† Tables S1 and S2). Moreover, both ADCs were well tolerated up to 20 mg kg^−1^. No statistically significant changes to mouse bodyweight were observed between any of the treatment arms compared to trastuzumab or vehicle, with bodyweight generally increasing throughout the study (Fig. 3c). No changes in bodyweight indicates that the ADCs were not causing systemic toxicity. Furthermore, no clinical signs of adverse effects were observed in any animals for the study duration.

**Fig. 3:**
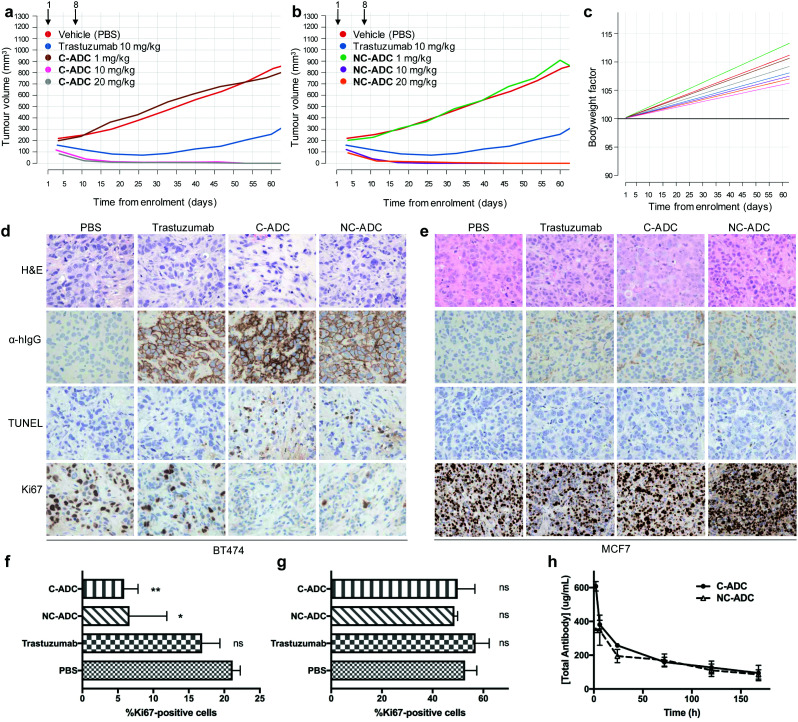
*In vivo* evaluation of DVP ADCs. Effect of BT474 cell line xenograft in NSG mice (*n* = 3) up to 60 days post-treatment with (a) C-ADC and (b) NC-ADC. (c) Changes in mouse bodyweight up to 60 days post-treatment with C-ADC or NC-ADC. H&E staining, anti-human IgG (α-hIgG), TUNEL and Ki67 IHC of (d) BT474 and (e) MCF7 mouse xenograft tumours 72 hours post-treatment with C-ADC, NC-ADC, trastuzumab or vehicle (PBS). Quantification of Ki67 levels from IHC analysis of (f) BT474 and (g) MCF7 mouse xenograft tumours (*n* = 3) 72 hours post-treatment with C-ADC, NC-ADC, trastuzumab or vehicle (PBS). Statistical significance calculated using a two-tailed paired *t* test by comparison to PBS treated animals. Not significant (ns) *p* > 0.05, *p < 0.05, **p < 0.005. (h) Pharmacokinetic analysis of the *in vivo* mouse plasma half-life of NC-ADC and C-ADC.

A significant reduction in tumour volume was observed 3–4 days after administration of the first dose of ADC; therefore, we next wanted to evaluate the molecular characteristics of the tumours within 72 hours of treatment. Accordingly, NSG mice bearing BT474 subcutaneous tumours were administered a single IV dose of vehicle or 10 mg kg^−1^ of trastuzumab, C-ADC or NC-ADC and tumours collected 72 hours later. H&E analysis suggested that tumours from all animals consisted predominantly of cancer cells while immunohistochemistry (IHC) displayed an enrichment of trastuzumab, NC-ADC or C-ADC at the cell surface of cancer cells (Fig. 3d). IHC further indicated that mice treated with either ADC had tumours with significantly lower levels of Ki67 (proliferation marker) compared to vehicle or trastuzumab treated animals (Fig. 3d and f). Conversely, ADC treated tumours had a higher TUNEL (apoptotic cell death) signal than control animals (Fig. 3d). To confirm that these results were due to the selective HER2 targeting ability of the ADCs, NSG mice bearing subcutaneous MCF7 tumours were subjected to the same single dose administration of vehicle, trastuzumab, C-ADC or NC-ADC. IHC analysis of tumours collected 72 hours after treatment indicated minimal localisation of trastuzumab or either ADC in these tumours (Fig. 3e). ADC or control treated tumours had near identical Ki67 and TUNEL staining profiles (Fig. 3e and g). Finally, H&E analysis of organs (brain, liver, kidney, small intestine, lung) suggested that the ADCs were not having significant effect on any of these tissues (ESI,† Fig. S4 and S5). These data suggest that our ADCs are efficacious and well tolerated *in vivo*, due to their highly selective toxicity for HER2-positive cells.

The exposure levels of an ADC correlate with anti-tumour efficacy and tolerability. To better understand the exposure levels of the ADCs, a single dose pharmacokinetic study in NSG mice treated with C-ADC or NC-ADC at 10 mg kg^−1^ was conducted (ESI,† Fig. S6). The results show that both ADCs had good exposure levels with *C*_max_ of 4.18 μmol L^−1^ AUC_0-inf_ of 328 μmol h^−1^ L^−1^ and blood half-life of 128 hours measured for the C-ADC (Fig. 3h). The NC-ADC had slightly lower exposure levels with *C*_max_ of 2.39 μmol L^−1^ AUC_0-inf_ of 262 μmol h^−1^ L^−1^ and blood half-life of 97 hours (Fig. 3h). For both ADCs, the linker-drug that would be released upon deconjugation was not observed in any animals.

This work represents the first *in vivo* proof of concept that DVP reagents can be used to generate safe and efficacious ADCs. A thorough structural characterisation revealed that these reagents generate the desired DAR 4 ADCs, with minor amounts of other DAR species present. The ADCs retained binding affinity to their target receptor and showed a wide *in vitro* therapeutic window when tested against a panel of cancer cell lines. Finally, the ADCs demonstrated exquisitely selective efficacy and tolerability in breast cancer xenograft models. This work highlights the utility of DVP reagents and validates the use of these molecules for the development of future ADCs.

The Spring Laboratory acknowledges support from the EPSRC, BBSRC, MRC and Royal Society. Research in the Carroll lab was funded by CRUK core funding awarded to JSC.

## Conflicts of interest

There are no conflicts to declare.

## Supplementary Material

CC-058-D1CC06766D-s001
