# Supplementary material for: Divinylpyrimidine reagents generate antibody–drug conjugates with excellent in vivo efficacy and tolerability
Source: Chem Commun (Camb). 2022 Jan 13;58(12):1962–5. doi: 10.1039/d1cc06766d (PMC9073851; doi:10.1039/d1cc06766d)
Supplement: CC-058-D1CC06766D-s001 [file CC-058-D1CC06766D-s001.pdf]

Electronic Supplementary Material (ESI) for ChemComm.
This journal is © The Royal Society of Chemistry 2022

Divinylpyrimidine reagents generate antibody-drug conjugates with excellent in vivo efficacy and tolerability

Stephen J. Walsh,[a,b] Soleilmane Omarjee,[b] Friederike M. Dannheim,[a] Dominique-Laurent Couturier,[b] Dorentina Bexheti,[b] Lee Mendil,[b] Gemma Cronshaw,[b] Toby Fewster,[b] Charlotte Gregg,[b] Cara Brodie,[b] Jodi L. Miller,[b] Richard Houghton,[b] Jason S. Carroll,[b]* and David R. Spring[a]*

[a] Yusuf Hamied Department of Chemistry, University of Cambridge, Lensfield Road, Cambridge, CB2 1EW, UK. Email: spring@ch.cam.ac.uk

[b] Cancer Research UK Cambridge Institute, University of Cambridge, Robinson Way, Cambridge, CB2 0RE, UK. Email: Jason.carroll@cruk.cam.ac.uk

Electronic Supporting Information

1. Chemical Synthesis 2
2. ADC Synthesis 7
3. Hydrophobic Interaction Chromatography (HIC) 9
4. Size Exclusion Chromatography (SEC) 10
5. Liquid Chromatography-Mass Spectrometry (LCMS) 10
6. Enzyme-linked Immunosorbent Assay (ELISA) 10
7. Cell Lines 11
8. In vitro Cell Viability Study 11
9. In vivo Efficacy Study 11
10. In vivo Selectivity Study 14
11. Pharmacokinetics Study 16
12. References 19

1. Chemical Synthesis

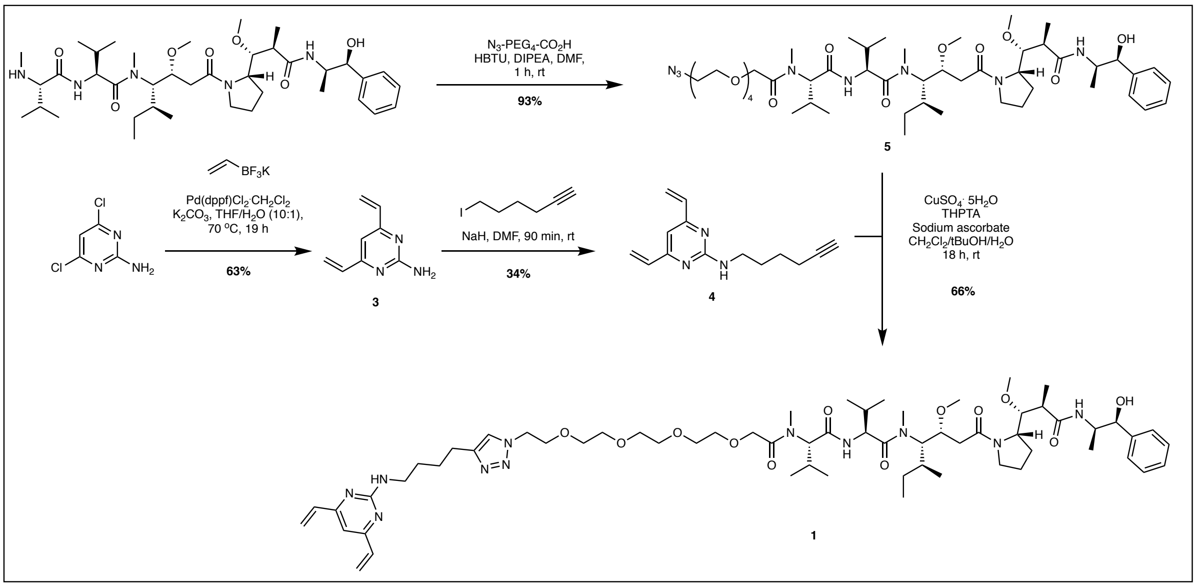

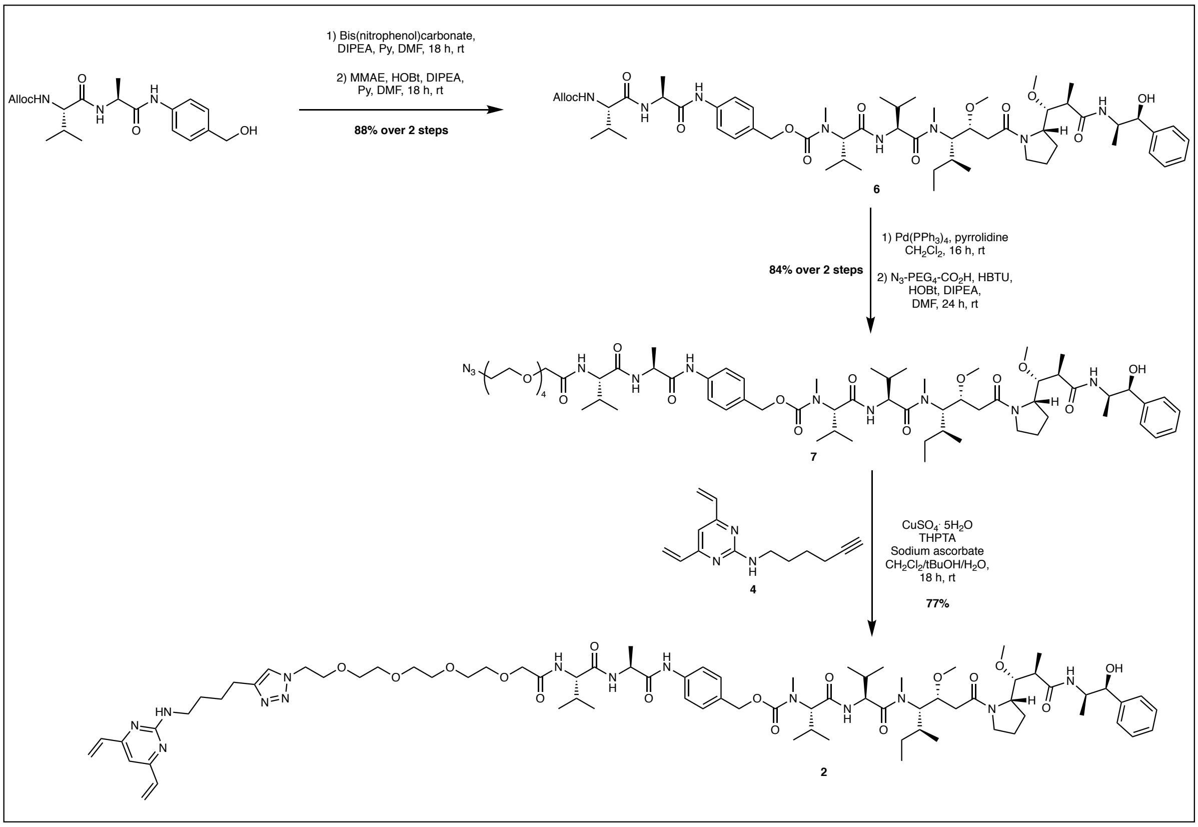

2-amino-4,6-divinylpyrimidine (3)

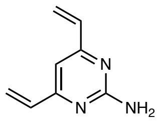

A solution of 2-amino-4,6-dichloropyrimidine (400 mg, 2.44 mmol), potassium vinyltrifluoroborate (980 mg, 7.32 mmol), Pd(dppf)$Cl_2 \cdot CH_2Cl_2$ (199 mg, 0.244 mmol) and potassium carbonate (2.02 g, 14.6 mmol) in THF/H_2O (10:1, 7.7 mL) was heated to 70 °C for 18 h. Upon completion, the reaction mixture was filtered through Celite® and the solvent removed *in vacuo*. The resulting residue was purified by FCC (10-40% EtOAc/PE) to yield 2-amino-4,6-divinylpyrimidine **3** (224 mg, 1.52 mmol, 63%) as an off-white solid.

R_f 0.37 (SiO_2; 50% EtOAc/PE); **δ_H** (400 MHz, CD_3OD) 6.79 (s, 1H), 6.61 (dd, 2H, *J* = 17.5, 10.8 Hz), 6.34 (dd, 2H, *J* = 17.5, 1.0 Hz), 5.63 (dd, 2H, *J* = 10.8, 1.0 Hz); **LRMS** (ESI) *m/z* found $[M+H]^+$ 148.2, $C_8H_{10}N_3^+$ required 148.1.

Data in accordance with literature.[1]

***N*-(hex-5-yn-1-yl)-4,6-divinylpyrimidin-2-amine (4)**

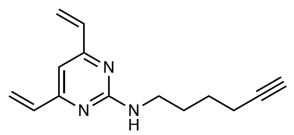

To a solution of amine **3** (50.0 mg, 0.340 mmol) in DMF (1.5 mL) was added 6-iodo-1-hexyne (54.0 µL, 0.410 mmol) followed by the slow addition of sodium hydride (60% in mineral oil, 16.4 mg, 0.410 mmol). The reaction mixture was stirred at rt for 90 min, then diluted with H_2O (15 mL) and extracted with CH_2Cl_2 (3 × 20 mL). The combined organic fractions were dried ($MgSO_4$) and concentrated *in vacuo*. The crude residue was purified by FCC (0-20% EtOAc/PE) to yield *N*-(hex-5-yn-1-yl)-4,6-divinylpyrimidin-2-amine **4** (26.1 mg, 0.115 mmol, 34%) as a clear oil.

R_f 0.39 (SiO_2; 25% EtOAc/PE); **δ_H** (400 MHz, $CDCl_3$) 6.59 (dd, 2H, *J* = 17.5, 10.7 Hz), 6.53 (s, 1H), 6.38 (d, 2H, *J* = 17.5 Hz), 5.58 (dd, 2H, *J* = 10.7, 1.2 Hz), 5.32 (s, 1H), 3.51 (q, 2H, *J* = 6.6 Hz), 2.23 (td, 2H, *J* = 10.5, 2.7 Hz), 1.95 (t, 1H, *J* = 2.7 Hz), 1.79-1.71 (m, 2H), 1.66-1.61 (m, 2H); **LRMS** (ESI) *m/z* found $[M+H]^+$ 228.3, $C_{14}H_{18}N_3^+$ required 228.1.

Data in accordance with literature.[1]

N_3-PEG$_4$-MMAE (5)

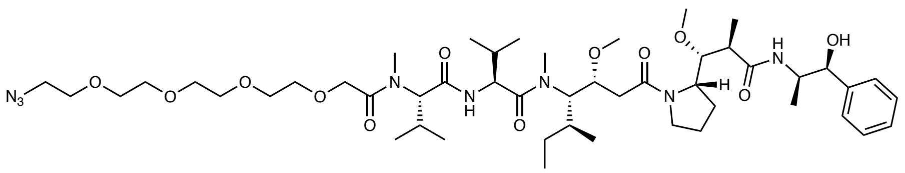

To a solution of N_3-PEG$_4$-COOH (93.0 µL, 42.0 µmol, 0.5 M in TBME, 90%), HBTU (21.2 mg, 56.0 µmol), HOBt (7.10 mg, 42.0 µmol) and DIPEA (29.3 µL, 168 µmol) in DMF (1.0 mL) was added a solution of MMAE (20.0 mg, 28.0 µmol) in DMF (0.5 mL) and the reaction mixture stirred at rt for 21 h. Upon completion, the solvent was removed under a stream of N_2 and the crude residue was purified by FCC (0-10% MeOH/CH_2Cl_2) to yield N_3-PEG$_4$-MMAE **5** (25.0 mg, 26.0 µmol, 93%) as a clear oil.

R$_f$ 0.5 (SiO_2; 10% MeOH/CH_2Cl_2); **HPLC** (5-95% MeCN/H_2O over 15 min) retention time 10.287 min; **LRMS** (ESI) *m/z* found $[M+H]^+$ 978.3, $C_{49}H_{85}N_8O_{12}^+$ required 977.6.

Data in accordance with literature.[1]

DVP-PEG$_4$-MMAE (1)

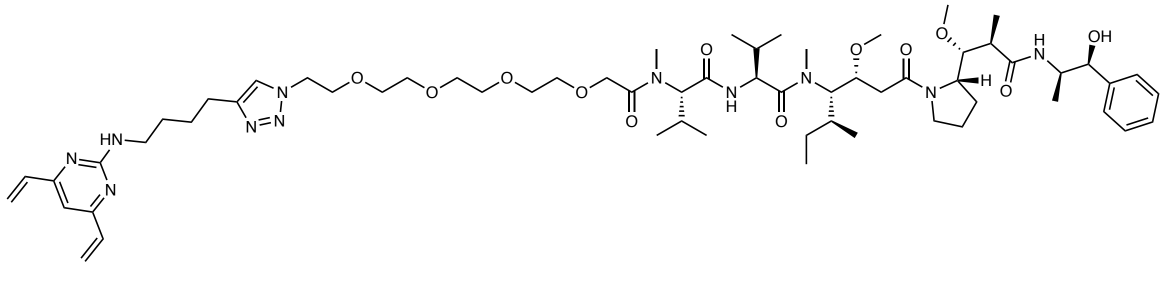

To a degassed solution of azide **5** (23.0 mg, 24.0 µmol) and alkyne **4** (10.9 mg, 48.0 µmol) in CH_2Cl_2 (0.5 mL) was added a degassed solution of $CuSO_4{\cdot}5H_2O$ (7.10 mg, 28.0 µmol), THPTA (20.9 mg, 48.0 µmol) and sodium ascorbate (23.8 mg, 120 µmol) in H_2O/^{t}BuOH (1 mL, 1:1) and the reaction mixture stirred at rt for 18 h. Upon completion, the reaction was diluted with H_2O (15 mL) and extracted with CH_2Cl_2 (4 × 20 mL). The combined organic fractions were dried ($MgSO_4$), concentrated *in vacuo* and the crude residue purified by FCC (0-10% MeOH/CH_2Cl_2) to yield DVP-PEG$_4$-MMAE **1** (19.0 mg, 15.8 µmol, 66%) as a clear oil.

R_f 0.29 (SiO_2; 7.5% MeOH/CH_2Cl_2); **HPLC** (5-95% MeCN/H_2O over 15 min) retention time 10.364 min; **LRMS** (ESI) *m/z* found $[M+H]^+$ 1205.8, $C_{63}H_{102}N_{11}O_{12}^+$ required 1204.8.

Data in accordance with literature.[1]

Alloc-Val-Ala-PABA-PNP

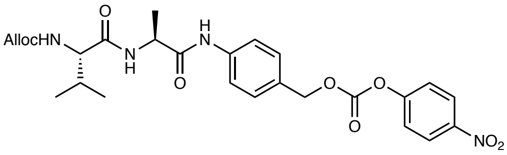

A solution of Alloc-Val-Ala-PABA (100 mg, 0.265 mmol), bis(4-nitrophenyl) carbonate (121 mg, 0.397 mmol) and DIPEA (231 µL, 1.33 mmol) in DMF (1 mL) was stirred at rt for 19 h. Upon completion, the mixture was concentrated under a stream of N_2. The crude residue was redissolved in CH_2Cl_2 (40 mL) and saturated aqueous $NaHCO_3$ (25 mL), the layers separated and the aqueous phase extracted with further CH_2Cl_2 (3 × 40 mL). The combined organic fractions were dried ($MgSO_4$) and concentrated to yield Alloc-Val-Ala-PABA-PNP as a pale yellow solid, which was carried forward without further purification.

Alloc-Val-Ala-PABC-MMAE (6)

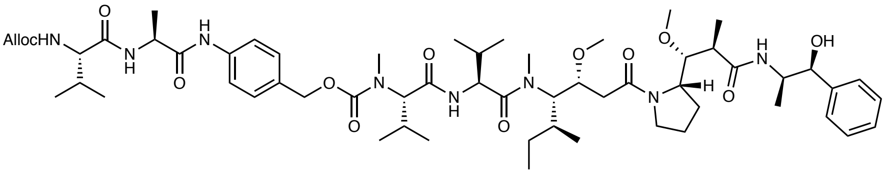

A solution of MMAE (26.5 mg, 37.0 µmol), Alloc-Val-Ala-PAB-PNP (30.0 mg, 55.0 µmol), HOBt (15.6 mg, 93.0 µmol), pyridine (36.8 µL, 455 µmol) and DIPEA (12.9 µL, 74.0 µmol) in DMF (0.5 mL) was stirred at rt for 18 h. Upon completion, the reaction mixture was concentrated under a stream of N_2 and the crude residue purified by FCC (0-6% MeOH/CH_2Cl_2) to yield Alloc-Val-Ala-PABC-MMAE **6** (36.4 mg, 32.5 µmol, 88%) as a white solid.

R_f 0.47 (SiO_2; 10% MeOH/CH_2Cl_2); **HPLC** (5-95% MeCN/H_2O over 15 min) retention time 12.380 min; **LRMS** (ESI) *m/z* found $[M+H]^+$ 1123.0, $C_{59}H_{93}N_8O_{13}^+$ required 1121.7.

Data in accordance with literature.[2]

N_3-PEG$_4$-Val-Ala-PABC-MMAE (7)

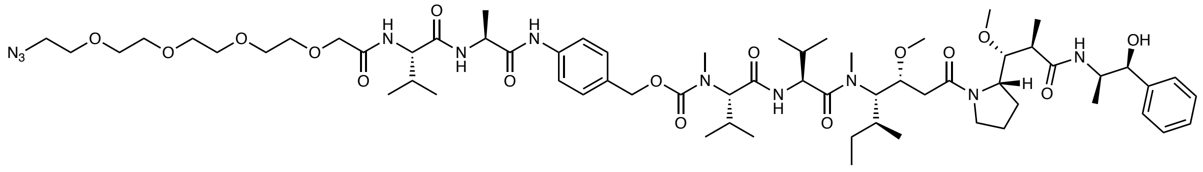

A solution of Alloc-Val-Ala-PABC-MMAE **6** (45.0 mg, 40.0 µmol) and $Pd(PPh_3)_4$ (2.30 mg, 2.00 µmol) and pyrrolidine (6.70 µL, 80.0 µmol) in CH_2Cl_2 (0.7 mL) was stirred at rt for 16 h. Upon completion, the reaction mixture was diluted with CH_2Cl_2 (15 mL) and saturated aqueous $NaHCO_3$ (15 mL). The layers were separated and the aqueous phase was extracted with further CH_2Cl_2 (3 × 20 mL). The combined organic fractions were dried ($MgSO_4$), concentrated *in vacuo* and the crude amine carried through without further purification.

A solution of crude amine (20.0 mg, 19.3 µmol), N_3-PEG$_4$-COOH (86.0 µL, 38.6 µmol, 0.5 M in TBME, 90%), HBTU (14.6 mg, 38.6 µmol), HOBt (6.50 mg, 38.6 µmol) and DIPEA (6.70 µL, 38.6 µmol) in DMF (1 mL) was stirred at rt for 24 h. Upon completion, the reaction mixture was diluted aqueous Na_2CO_3 (1 M, 15 mL) and extracted with CH_2Cl_2 (4 × 15 mL). The combined organic fractions were dried ($MgSO_4$), concentrated *in vacuo* and the crude residue was purified by FCC (0-8% MeOH/CH_2Cl_2) to yield N_3-PEG$_4$-Val-Ala-PABC-MMAE **7** (21.1 mg, 16.3 µmol, 84%) as a clear oil.

R_f 0.28 (SiO_2; 10% MeOH/CH_2Cl_2); **HPLC** (5-95% MeCN/H_2O over 20 min) retention time 12.011 min; **LRMS** (ESI) *m/z* found $[M+H]^+$ 1298.1, $C_{65}H_{106}N_{11}O_{16}^+$ required 1296.8.

Data in accordance with literature.[2]

DVP-PEG$_4$-Val-Ala-PABC-MMAE (2)

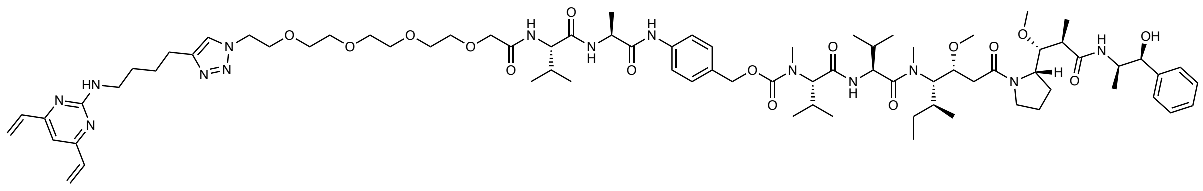

To a degassed solution of azide **7** (18.0 mg, 13.9 µmol) and alkyne **4** (6.30 mg, 27.8 µmol) in CH_2Cl_2 (0.5 mL) was added a degassed solution of $CuSO_4{\cdot}5H_2O$ (4.20 mg, 16.7 µmol), THPTA (12.1 mg, 27.8 µmol) and sodium ascorbate (13.8 mg, 69.5 µmol) in $H_2O/^tBuOH$ (1 mL, 1:1) and the reaction mixture stirred at rt for 18 h. Upon completion, the reaction was diluted with H_2O (15 mL) and extracted with CH_2Cl_2 (4 × 20 mL). The combined organic fractions were dried ($MgSO_4$), concentrated *in vacuo* and the crude residue purified by FCC (0-8% MeOH/CH_2Cl_2) to yield DVP-PEG$_4$-Val-Ala-PABC-MMAE **2** (16.4 mg, 10.8 µmol, 77%) as a pale yellow oil.

R$_f$ 0.22 (SiO_2; 10% MeOH/CH_2Cl_2); **HPLC** (5-95% MeCN/H_2O over 20 min) retention time 11.027 min; **LRMS** (ESI) *m/z* found $[M+H]^+$ 1525.1, $C_{79}H_{123}N_{14}O_{16}^+$ required 1523.9.

Data in accordance with literature.[2]

2. ADC Synthesis

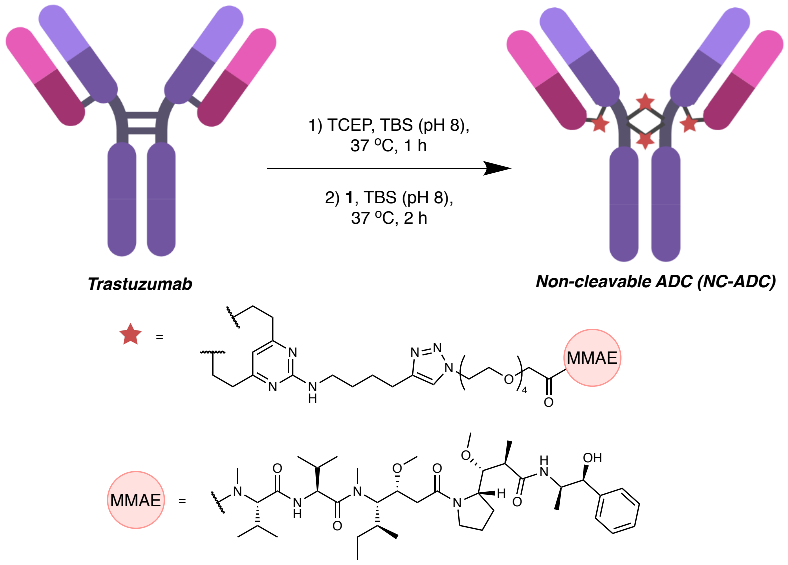

To a solution of trastuzumab (900 µL, 20.41 µM, 3.0 mg/mL) in TBS (25 mM Tris HCl pH 8, 25 mM NaCl, 0.5 mM EDTA) was added TCEP (10 equiv.). The mixture was vortexed and incubated at 37 °C for 1 h. A solution of DVP **1** (20 mM in DMSO, adjusted to 10% v/v) was added (40 equiv.) and the reaction mixture incubated at 37 °C for 2 h. The excess reagents

were removed by two successive PD-10 columns (GE Healthcare) pre-equilibrated with PBS. The reaction product was completely buffer exchanged into PBS and concentrated by repeated diafiltration using an Amicon-Ultra centrifugal filter (10,000 MWCO, Merck Millipore).

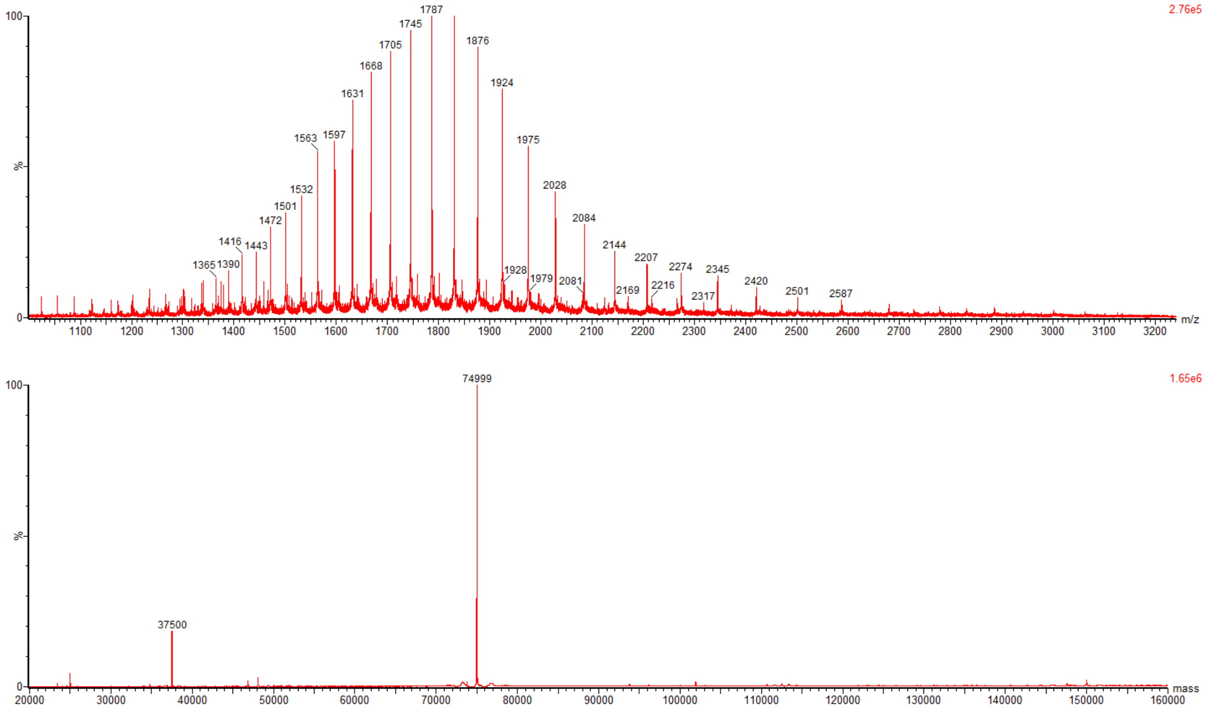

Fig. S1: LCMS analysis of **NC-ADC**. The raw mass spectrum (top) and deconvoluted mass spectrum (bottom) are shown.

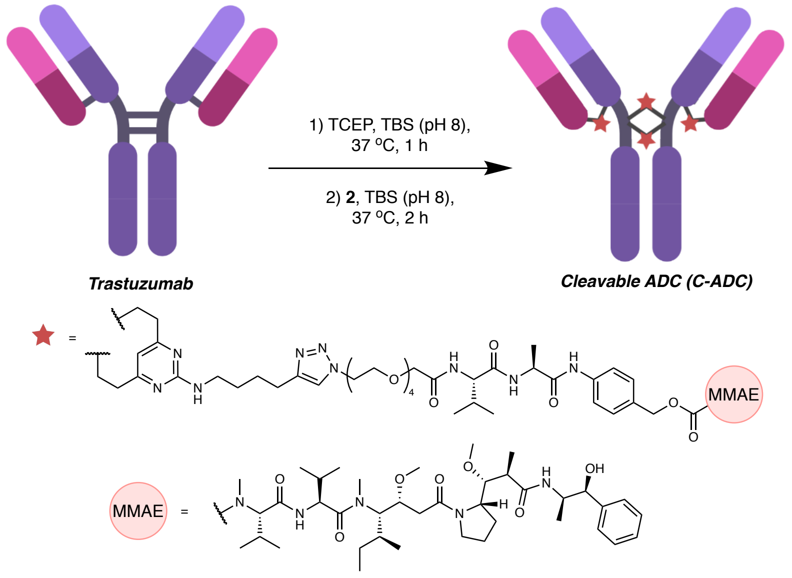

To a solution of trastuzumab (900 µL, 20.41 µM, 3.0 mg/mL) in TBS (25 mM Tris HCl pH 8, 25 mM NaCl, 0.5 mM EDTA) was added TCEP (10 equiv.). The mixture was vortexed and incubated at 37 °C for 1 h. A solution of DVP **2** (20 mM in DMSO, adjusted to 10% v/v) was

added (40 equiv.) and the reaction mixture incubated at 37 °C for 2 h. The excess reagents were removed by two successive PD-10 columns (GE Healthcare) pre-equilibrated with PBS. The reaction product was completely buffer exchanged into PBS and concentrated by repeated diafiltration using an Amicon-Ultra centrifugal filter (10,000 MWCO, Merck Millipore).

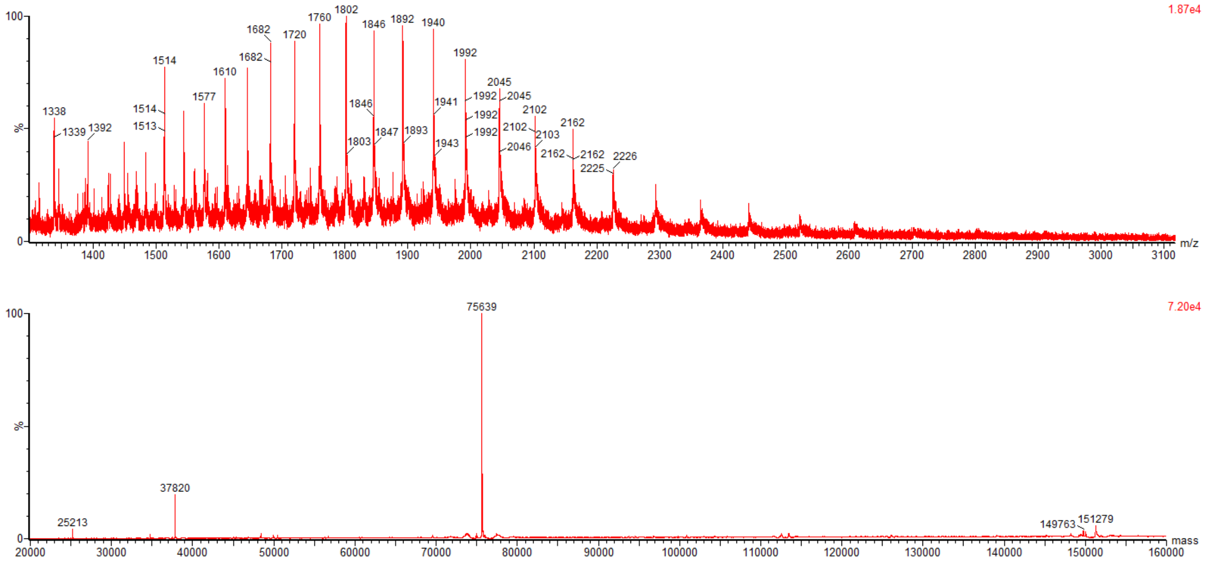

Fig. S2: LCMS analysis of **C-ADC**. The raw mass spectrum (top) and deconvoluted mass spectrum (bottom) are shown.

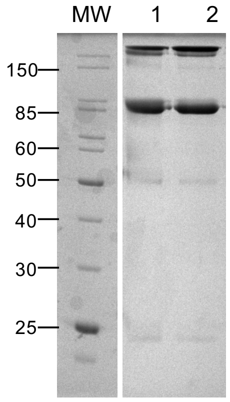

Fig. S3: Reducing SDS-PAGE analysis of **NC-ADC** and **C-ADC**. MW = molecular weight ladder, lane 1 = **NC-ADC**, lane 2 = **C = ADC**.

3. Hydrophobic Interaction Chromatography (HIC)

Hydrophobic interaction chromatography (HIC) was carried out on an Agilent 1200 Series system using a Tosoh Bioscience TSKgel Butyl-NPR (3.5 cm × 4.6 mm, 2.5 μm particle size) column at a flow rate of 0.6 mL/min and a gradient of 0-100% B over 20 minutes (Solvent A: 1.5M ammonium sulfate, 25 mM NaPi, pH 7; Solvent B: 25% isopropyl alcohol in 25 mM NaPi, pH 7). 10 μg of trastuzumab or ADC (1-2 mg/mL in PBS) was analysed per run. Average DAR for each ADC was calculated as follows, where DAR_n corresponds to the peak area at 280 nm

for a given DAR species, with n representing the number of DVP-MMAE molecules per antibody for that DAR species.

$$Average\ DAR = \frac{(DAR_1 + 2 \times DAR_2 + 3 \times DAR_3 + 4 \times\ DAR_4 + 5 \times DAR_5)}{(DAR_0 + DAR_1 + DAR_2 + DAR_3 + DAR_4 + DAR_5)}$$

4. Size Exclusion Chromatography (SEC)

Size-exclusion chromatography (SEC) was carried out on an Agilent 1200 Series system using a TSKgel G3000SWXL (30 cm × 7.8 mm, 5 μm particle size) with a mobile phase of PBS (50 mM sodium phosphates, 100 mM NaCl, 0.02% sodium azide, pH 7.0) at a flow rate of 0.5 mL/min over 30 min. 10 μg of trastuzumab or ADC (1-2 mg/mL in PBS) was analysed per run. Samples were analysed via absorption at 280 nm.

5. Liquid Chromatography-Mass Spectrometry (LCMS)

Protein LCMS was performed on a Xevo G2-S TOF mass spectrometer coupled to an Acquity UPLC system using an Acquity UPLC BEH300 C4 column (1.7 μm, 2.1 × 50 mm). H_2O with 0.1% formic acid (solvent A) and 95% MeCN and 5% water with 0.1% formic acid (solvent B), were used as the mobile phase at a flow rate of 0.2 mL/min. The gradient was programmed as follows: 95% A for 0.93 min, then a gradient to 100% B over 4.28 min, then 100% B for 1.04 minutes, then a gradient to 95% A over 1.04 min. The electrospray source was operated with a capillary voltage of 2.0 kV and a cone voltage of 150 V. Nitrogen was used as the desolvation gas at a total flow of 850 L/h. Total mass spectra were reconstructed from the ion series using the MaxEnt algorithm preinstalled on MassLynx software (v4.1 from Waters) according to the manufacturer's instructions. Trastuzumab samples were deglycosylated with PNGase F (New England Biolabs) prior to LCMS analysis.

6. Enzyme-linked Immunosorbent Assay (ELISA)

A 96-well plate was coated with 100 µL of a 0.25 µg/mL solution of HER2 (Sino Biological, His-tagged) overnight at 4 °C. Coating solutions were removed and each well washed with PBS (2 × 200 µL). Each well was then blocked with 1% BSA in PBS (200 µL) for 1 h at room temperature. The blocking solution was then removed and each well washed with PBS (3 × 200 µL). Wells were treated with a serial dilution of trastuzumab and ADCs in PBS (100 µL of 30 nM, 10 nM, 3.33 nM, 1.11 nM, 0.37 nM, 0.12 nM, 0 nM) and incubated at room temperature for 2 h. The conjugate solutions were removed and each well was washed with 0.1% Tween 20 in PBS (2 × 200 µL) followed by PBS (3 × 200 µL). Next, 100 µL of detection

antibody (1:1000 dilution of a mouse anti-human IgG-HRP, ThermoFisher) in PBS was added to each well and incubated at room temperature for 1 h. Each well was washed with 0.1% Tween 20 in PBS (2 × 200 µL) followed by PBS (3 × 200 µL). Finally, an OPD solution (100 µL of a solution prepared by dissolving 1 capsule in 9 mL H_2O and 1 mL stable peroxide substrate buffer (10×), ThermoFisher) was added to each well. After 10-15 minutes, 4M $HCl_{(aq)}$ (50 µL) was added to each well to quench the reaction. Absorbance was measured at 490 nm and 590 nm. Measurements were performed in quadruplicate and three independent repeats were performed.

7. Cell Lines

HER2-positive SKBR3 and BT474 cells were obtained from the American Type Culture Collection (ATCC) and HER2-negative MCF7 and MDA-MB-468 cells were obtained from the European Collection of Authenticated Cell Cultures (ECACC) and ATCC, respectively. SKBR3 cells were maintained in high glucose McCoy's 5A medium, supplemented with 10% heat-inactivated foetal-bovine serum (FBS), 50 U/mL penicillin and 50 µg/mL streptomycin. MCF7 and MDA-MB-468 cells were maintained in Dulbecco's Modified Eagle Medium (DMEM) supplemented with 10% FBS, 2 mM L-glutamine, 50 U/mL penicillin and 50 µg/mL streptomycin. BT474 cell lines were maintained in RPMI1640 medium supplemented with 10% FBS, 2 mM L-glutamine, 50 U/mL penicillin and 50 µg/mL streptomycin. All cell lines were incubated at 37 °C with 5% CO_2.

8. *In vitro* Cell Viability Study

Cells were seeded in 96-well plates for 24 h at 37 °C with 5% CO_2. SKBR3 cells were seeded at 15,000 cells/well, BT474 cells were seeded at 20,000 cells/well, MCF7 cells were seeded at 7,500 cells/well and MDA-MB-468 cells were seeded at 10,000 cells/well. Serial dilutions of trastuzumab, **C-ADC** and **NC-ADC** were added to the cells in complete growth medium and incubated at 37 °C with 5% CO_2 for 96 h. Cell viability was measured using CellTiter-Glo viability assay (Promega) according to the manufacturer's instructions. Cell viability was plotted as a percentage of untreated cells. Each measurement was taken in triplicate and three independent repeats were performed.

9. *In vivo* Efficacy Study

All animal experiments were carried out at the Biological Resource Unit at Cancer Research UK (CRUK) Cambridge Institute. The experiments were performed in accordance with the UK

Animals (Scientific Procedures) Act 1986, with approval from the CRUK Cambridge Institute Animal Ethical Review and Welfare Body. Female NSG mice (6-8 weeks old) were purchased from Charles River. The animals were verified as pathogen free and in excellent health. Subcutaneous BT474 xenografts were established by implantation of 10^7 cells in PBS/Matrigel (1:1, 200 μL). From the day of implantation, the drinking water was supplemented with β-estradiol (Sigma-Aldrich) (5 μg/mL, 0.1% ethanol). When tumour volumes reached 200 mm^3 (4-8 weeks after implantation), mice were randomised and enrolled to the study (*n* = 3 per study arm). Mice received two, weekly tail vein intravenous bolus injections of trastuzumab, **NC-ADC**, **C-ADC** or vehicle (PBS). Bodyweight and tumour volumes were measured 2-3 times per week for 60 days after enrolment. Tumour volume was measured using a Vernier caliper and was calculated as follows, where l is equal to the length (mm) of the longest tumour side and w is equal to the width (mm) of the shortest tumour side.

$$Tumour\ volume\ (mm^3) = \frac{l \times (2 \times w)}{2}$$

To model the tumour growth per mouse, we used a point-wise random intercept and slope linear mixed model (R function nlme::lme) with [i] the tumour volume on the cube root scale (to normalise data, linearise the relationship between time and growth and tame heteroscedasticy), [ii] mice as random effect, [iii] time from enrolment and group as fixed effects (with interaction terms allowing different growth rate per group). Furthermore, we allowed two inflection points (change in tumour growth rate) to occur between day 1 and 60 and estimated their location by minimising the residual sum of squares. Model checks suggested a good fit of the model to the data. Comparison of the tumour growth rates of interest (ESI Table S1) was performed with a multiplicity adjustment allowing to take the dependence between the contrasts of interest into account when setting the family-wise error rate at a 5% level (R function multcomp::glht).

Table S1: Statistical analysis of tumour growth rates.

Comparison	Statistic	Adjusted p-value	Sig.
PBS versus (anytime) vs Trastuzumab @ 10mg/kg (after day 25)	-2.1624866	0.1425	
**NC-ADC** @ 1 mg/kg (anytime) vs Trastuzumab @ 10mg/kg (after day 25)	-1.799431	0.2949	
**C-ADC** @ 1 mg/kg (anytime) vs Trastuzumab @ 10mg/kg (after day 25)	-2.3631143	0.0901	.
Trastuzumab @ 10mg/kg (after day 25) vs **NC-ADC** @ 10 mg/kg (after day 15)	11.3475626	<0.0001	***
Trastuzumab @10mg/kg (after day 25) vs **NC-ADC** @ 20 mg/kg (after day 15)	12.4725311	<0.0001	***
Trastuzumab @ 10mg/kg (after day 25) vs **C-ADC** @ 10 mg/kg (after day 15)	11.1183878	<0.0001	***
Trastuzumab @ 10mg/kg (after day 25) vs **C-ADC** @ 20 mg/kg (after day 15)	13.0361462	<0.0001	***

To compare the average tumour volume at day 60 of the different groups with respect to the reference one, 100 ul PBS, we first fitted a linear model (R function stats::lm) with the response on the cube root scale and 'group' as predictor, and then analysed the contrasts of interest (linear combinations of regression parameters, ESI Table S2.) using a Dunnett-type multiplicity correction allowing to take the dependence between the different comparisons of interest into account when setting the family-wise error rate at a 5% level (R function multcomp::glht). Sensitivity analyses considering heteroscedastic modelling led to the same conclusions.

Table S2: Statistical analysis of tumour volumes 60 days after study enrolment.

Comparison	Statistic	Adjusted p-value	Sig.
Trastuzumab @ 10mg/kg vs PBS	-5.112403161	0.0007	***
**NC-ADC** @ 1 mg/kg vs PBS	0.727636105	0.9622	
**NC-ADC** @ 10 mg/kg vs PBS	-7.343271533	<0.0001	***
**NC-ADC** @ 20 mg/kg vs PBS	-7.342875813	<0.0001	***
**C-ADC** @ 1 mg/kg vs PBS	-0.462630976	0.9967	
**C-ADC** @ 10 mg/kg vs PBS	-6.570414028	<0.0001	***
**C-ADC** @ 20 mg/kg vs PBS	-7.340337957	<0.0001	***

10. *In vivo* Selectivity Study

Female NSG mice (6-8 weeks old) were purchased from Charles River. The animals were verified as pathogen free and in excellent health. Subcutaneous BT474 xenografts were established by implantation of 10^7 cells in PBS/Matrigel (1:1, 200 μL). Subcutaneous MCF7 xenografts were established by implantation of 10^6 cells in PBS/Matrigel (1:1, 200 μL). From the day of implantation, the drinking water was supplemented with β-estradiol (Sigma-Aldrich) (5 μg/mL, 0.1% ethanol). When tumour volumes reached 200 mm^3, mice were randomised and enrolled to the study (*n* = 3 per study arm). Mice received a tail vein intravenous bolus injection of trastuzumab, **NC-ADC**, **C-ADC** or vehicle (PBS). 72 hours after treatment, mice were euthanised. Tumours and organs were excised and fixed with 10% neutral buffered formalin (NBF). Upon fixation, tissue samples were embedded in paraffin and stored for histology. H&E, TUNEL staining and Ki67 was carried out as standard. Trastuzumab (anti-human IgG) staining was performed by pre-treatment with Tris EDTA at 100 °C followed by probing with rabbit anti-human antibody (2.5 μg/mL, Abcam, ab98568). For quantification of Ki67 the slides were digitalised on an Aperio AT2 (Leica) at a resolution of 0.5 μm/pixel. HALO v3.2.1851.266 (Indica Labs) and the Multiplex IHC x2.3.4 with optical densities for weak, moderate and strongly stained nuclei set at 0.2102, 0.2876 and 0.708, respectively.

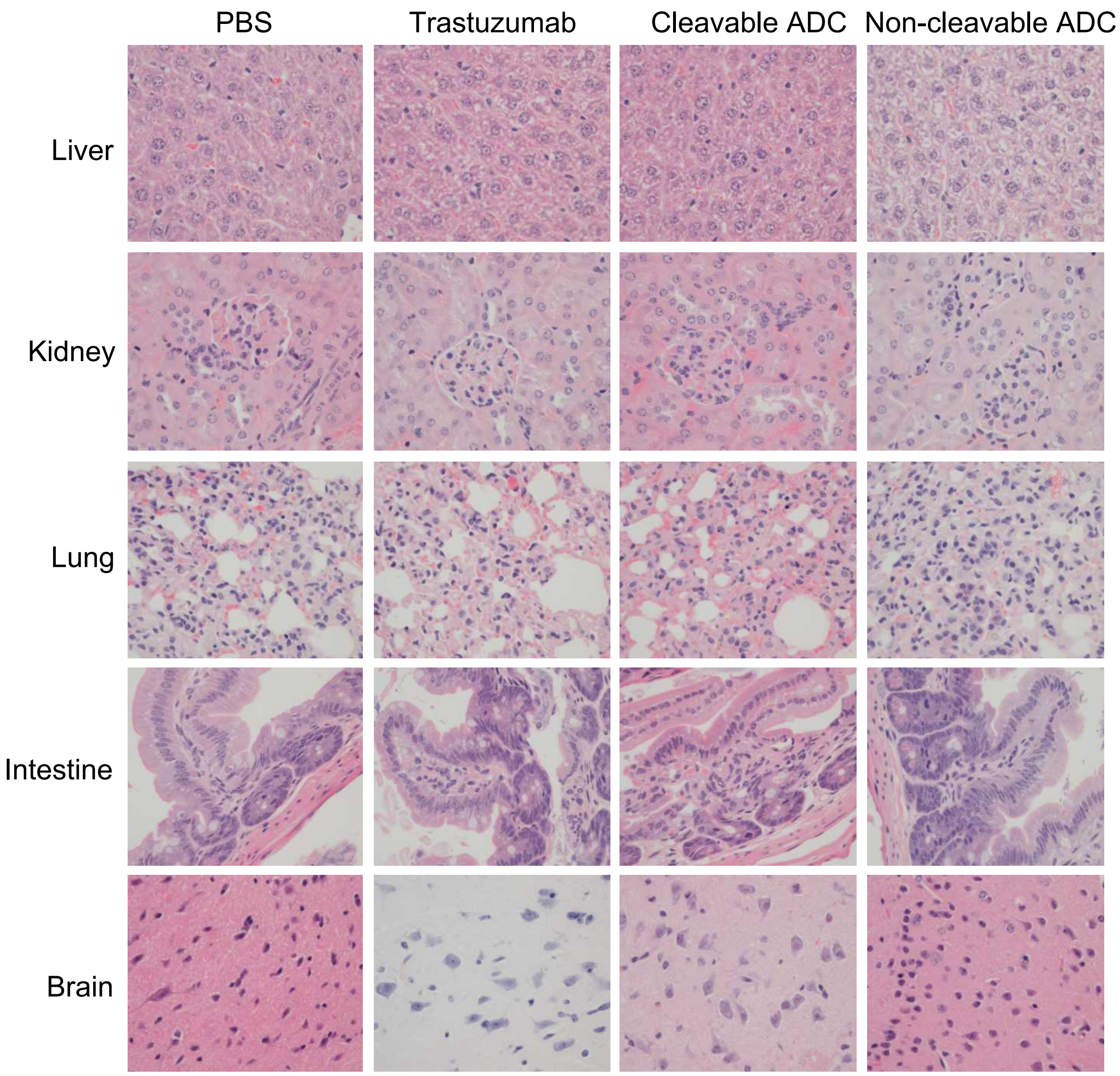

Fig. S4: H&E analysis of organs from animals containing BT474 cell xenografts.

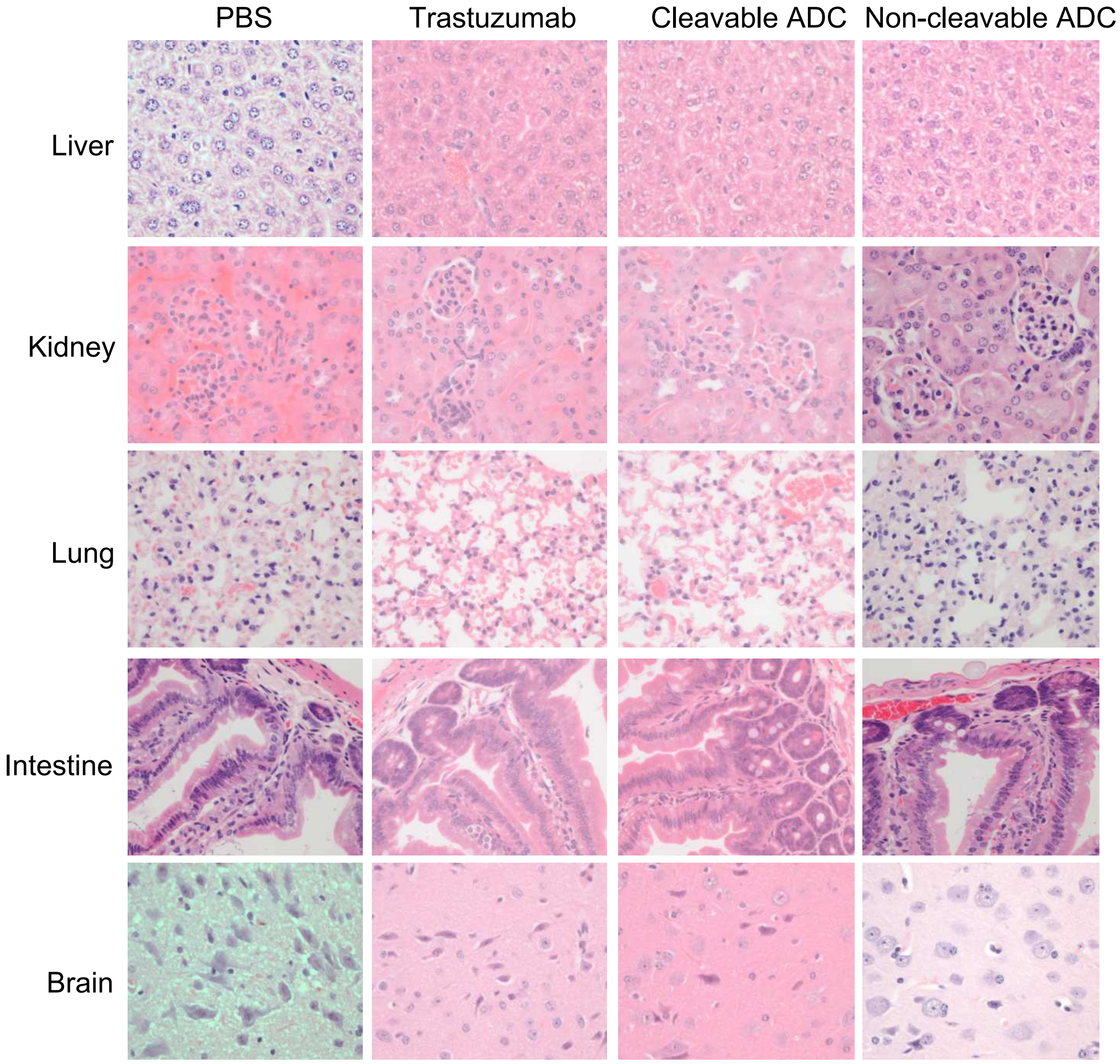

Fig. S5: H&E analysis of organs from animals containing MCF7 cell xenografts.

11. Pharmacokinetics Study

Female NSG mice (6-8 weeks old) were purchased from Charles River. The animals were verified as pathogen free and in excellent health. Subcutaneous BT474 xenografts were established by implantation of 10^6 cells in PBS/Matrigel (1:1, 200 μL). 7 days after implantation, mice were randomised and enrolled to the study (*n* = 3 per study arm). Mice received a tail vein intravenous bolus injection of **NC-ADC** or **C-ADC**. Blood samples were collected 2, 6, 24, 72, 120 and 168 hours after treatment. K_2EDTA (0.5 M in PBS) was added to each sample to a final concentration of 1.8 mg/mL of blood. Samples were stored on ice before centrifugation at 1600×g at 4 °C for 10 min. The plasma fraction was removed and stored at -80 °C until analysis.

A reference standard of Trastuzumab was purchased from Generon Ltd., Slough, UK. The signature peptide used for quantitation was H-(FTISADTSK)-OH and an isotopically labelled peptide H-GR(FTISADTSK*)-OH used as an internal standard (IS), both purchased from Pepscan, Lelystad, Netherlands. Optima-grade formic acid and acetonitrile were supplied by Fisher Scientific, UK. Guanidine hydrochloride was purchased from MP Biomedicals, UK, dithiothreitol (DTT) from GE healthcare, iodoacetamide from ACROS organics, ammonium bicarbonate from Sigma Aldrich, UK and Pierce trypsin protease (MS grade) from Thermo Fisher. Ultra-pure water (18.2 MΩ) was dispensed from Milli-Q systems, Merck Life Science UK Limited.

Stock solutions of Trastuzumab (6.53 mg/mL) were prepared in PBS and stored at -20 °C for up to 3 months. A calibration curve was prepared with Trastuzumab concentrations of 5, 10 , 25, 50, 80, 100, 200, 300, 400 & 600 µg/mL in control mouse plasma in Lobind tubes (Eppendorf UK Ltd., Stevenage). Quality control (QC) samples were prepared similarly (low: 30 µg /mL, medium: 80 µg/mL, high: 250 µg /mL & upper limit of quantitation: 500 µg /mL). IS concentration was commercially prepared at 1 mg/mL in PBS and diluted to a working concentration of 10 µg/mL in 50 mM ammonium bicarbonate solution.

10 µL aliquots of mouse plasma were taken for extraction by firstly precipitating the proteins using 40 µL of acetonitrile:0.1% formic acid (60:40, v/v), followed by centrifugation at 2000 g for 10 minutes. The sample supernatants were then discarded, and the pellets allowed to dry for 10 minutes in the tube, before resuspension in 20 µL of 6 M guanidine hydrochloride solution. 325 µL of 10mM DTT was then added, mixed well, and samples incubated for 90 minutes at 37 °C. 60 µL of 100 mM iodoacetamide solution was then added to the sample tubes, under dark room conditions, after which samples were left for 45 minutes at room temperature. 100 µL of the sample supernatant was then transferred into a new Eppendorf Lobind tube, 10 µL of internal standard working solutions added followed by 25 µL of 200 µL/mL trypsin solution. Sample were mixed well and left to incubate at 37 °C overnight (16-18 hours).

The enzyme reaction was halted by the addition of 35 µL of 1% formic acid (aq) solution followed by vortex mixing. Sample solutions were then transferred to a Lobind 96 well plate, centrifuged and injected into the LC-MS/MS instrument.

Concentrations of the signature peptide (FTISADTSK) were quantified using reversed phase ultra-high-performance liquid chromatography tandem mass spectrometry (RP-UHPLC-MS/MS). Instrumental analysis was performed on a Nexera X2 UHPLC (Shimadzu UK, Milton Keynes) coupled to a Sciex Triple Quad 6500 mass spectrometer (AB Sciex LLC, Framingham, USA). The prepared samples were injected onto a HSS T3, 1.8 µm, 100 x 2.1 mm analytical column (Waters, Wilmslow, UK) at 60 °C. Gradient elution was performed using 0.2% formic acid in water and an increasing percentage of 0.2% formic acid in acetonitrile at a flowrate 0.7 mL/min, with a total run time of 5.0 minutes. The mass spectrometer was operated using Analyst® v1.7.1 software in positive electrospray ionisation mode with MRM transitions of 485.30→721.37 (FTISADTSK) and 489.25⍰729.40 (IS).

The peak area ratios from the integrated analyte and internal standard chromatograms were used to back calculate the concentrations of Trastuzumab against a quadratic 1/x2 weighted calibration curve using MultiQuant™ v3.0.3 software (AB Sciex LLC, Framingham, USA).

Sample analysis was conducted in a batch, consisting of calibration standards, quality controls (QCs), blank control matrix samples, internal standard only (zero sample) and study samples. Precision and accuracy of the QCs were within the acceptance criteria of ±20%. Time versus concentration data was imported into PCModfit V6.0 software using a Microsoft Pharmacokinetic parameters were estimated using non-compartmental analysis.

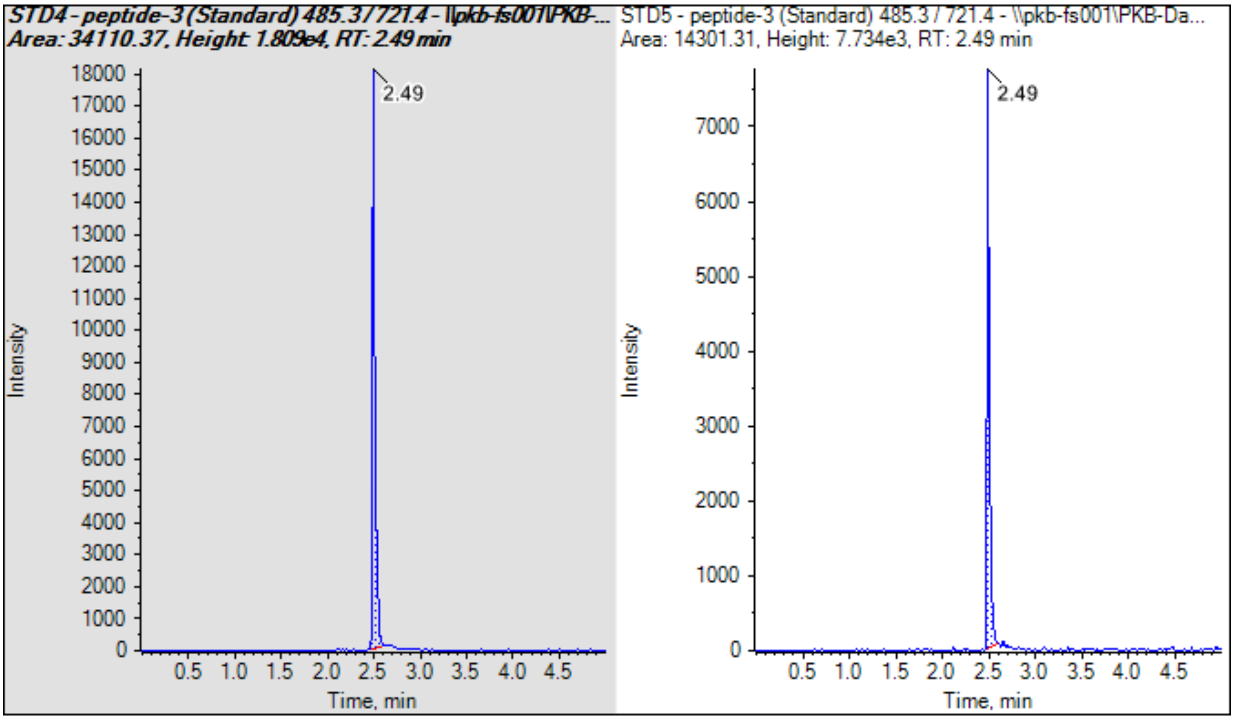

Fig. S6: Representative chromatograms for signature peptide (FTISADTSK) in calibration standards used for the quantification of trastuzumab in mouse plasma.

12. References

1 S. J. Walsh, S. Omarjee, W. R. J. D. Galloway, T. T.-L. Kwan, H. F. Sore, J. S. Parker, M. Hyvönen, J. S. Carroll and D. R. Spring, *Chem. Sci.*, 2019, **10**, 694–700.

2 J. D. Bargh, S. J. Walsh, A. Isidro-Llobet, S. Omarjee, J. S. Carroll and D. R. Spring, *Chem. Sci.*, 2020, **11**, 2375–2380.
